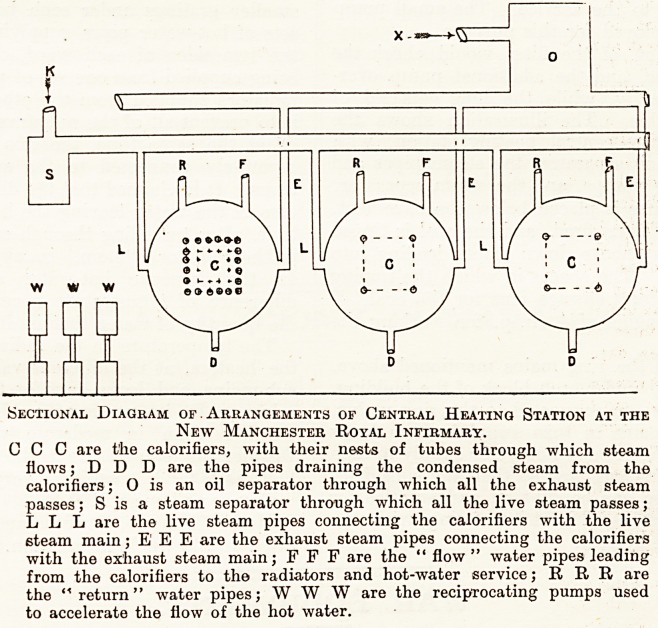# The Manchester Royal Infirmary

**Published:** 1915-10-16

**Authors:** 


### THE HEATING OF HOSPITALS.*

**[NOTE.—In a series of articles the writer proposes to describe the different systems of heating that are employed in hospitals. He will be pleased to answer any questions through "The Hospital," bearing upon the subject of heating or ventilation. Each system will be illustrated and described as exemplified at some hospital where it has been applied.**

### IV. The Manchester Royal Infirmary.

ACCELERATED HOT-WATER SYSTEM.

At the Manchester Royal Infirmary the same ideas with regard to economy underlie the whole installation as at Leicester, but they are worked out in quite a different manner.

The whole of the exhaust steam from the engines and the condensed water from the various heating appliances, the steam pipes, etc., are brought to a central heating station. The heating plant consists of three Lancashire boilers, each 28 ft. long by 8 ft. in diameter, all working at 70 lbs. pressure. The central heating station is placed close to the boiler room. All of the exhaust steam is brought to an oil separator fixed in the central heating station, and it passes from the separator to one of the calorifiers, or, as the makers in this case prefer to call them, "patent heaters." There are three heaters, each consisting of an outer cylinder in which the water to be heated circulates, with a nest of tubes on the inside through which the steam employed in heating the water passes. The nest of tubes can be withdrawn for cleaning purposes. One of the heaters is used for the hot-water domestic service, another for the hot water used in the radiators. The third heater is kept as a standby, and can be employed for either heating the domestic hot-water supply or the hot water used in the radiators. All three of the heaters are arranged to take exhaust steam from the oil separator, or live steam directly from the boiler.

There are two sets of steam pipes in the central heating station, one supplying live steam, the other exhaust steam, both pipes being connected by valves to all the heaters. There is a hot well placed under the heating station into which the condensed steam from the heaters and from all the other heating appliances about the building, from the steam traps, etc., is led. There are three reciprocating pumps in the central heating station, one pump for each of the heaters. Each pump is arranged to be connected to the service supplied by either heater.

The reciprocating pumps give the accelerated flow to the hot water both for the radiators and for the domestic supply that forms the principal feature of the system. The increased velocity at which the hot water flows through the system allows smaller pipes to be employed for both services, and, it is claimed, gives rise to economical working generally. The distribution of hot water, both for the domestic supply and the radiators, is by means of two pairs of ring mains running round the subways. One pair of mains supplies the radiators, and the other pair the hot water for domestic purposes. The two pairs of mains are also interconnected by systems of valves, so that either pair can be used for each purpose; the chances of the supply of either hot water or heat in the radiators failing being thus reduced to a minimum.

Sectional Diagram of Arrangements of Central Heating Station at the New Manchester Royal Infirmary.

C C C are the calorifiers, with their nests of tubes through which steam flows; D D D are the pipes draining the condensed steam from the calorifiers; O is an oil separator through which all the exhaust steam passes; S is a steam separator through which all the live steam passes; L L L are the live steam pipes connecting the calorifiers with the live steam main; E E E are the exhaust steam pipes connecting the calorifiers with the exhaust steam main; F F F are the "flow" water pipes leading from the calorifiers to the radiators and hot-water service; R R R are the "return" water pipes; W W W are the reciprocating pumps used to accelerate the flow of the hot water.

In the event of trouble arising in any portion of either of the ring mains, that length can be cut off, the supply being maintained by the others while the trouble is being put right. The water used in the hot-water service, and any leakage that may take place from the pipes supplying the radiators, is made up from two tanks of 6,000 gallons each in the tower. The tanks contain town's water; and it is led to the suction pipe of the heater supplying the domestic hot-water service by gravity.

The boiler is fed from the hot well, there being duplicate feed pumps and duplicate feed pipes. There are two steam separators on the main steam

* Previous articles appeared on July 31, September 11, and October 2, 1915.

pipes leading to the engine. In addition to the reciprocating pumps mentioned above, giving what the makers term acceleration to the water, there is a smaller pump which is what the Americans call a "booster."

### "Hardening" the Water.

As is well known, the water in Manchester is very soft, and it has been found that the insides of the pipes rust very quickly if the water is used in either the domestic hot-water system or the radiator system without treatment. The trouble is so great that the iron rust was stated to the writer to close up the pipes in about three months. To meet the difficulty lime is added to the water in certain proportions sufficient to give 7° of hardness, and the water so treated is forced through a filter on its way to the service. The small pump mentioned is employed for this purpose.

The introduction of the filter would check the flow of the water, and the additional pump overcomes that difficulty, while the lime gets rid of the rusting trouble. The illustration shows the arrangement of the central heating station, with the heaters, the oil separator, the steam pipes and the reciprocating pumps, and the steam separator.

The boiler house is placed below the pavement, the furnaces are hand fired, and the coal is tipped from the roadway above down slopes leading into the boiler house. The spaces in which the tipping slopes are fixed form storage bins for the coal.

There is an engine of 40 h.-p. for working the laundry.

In addition to the ring mains mentioned above, which are carried under each block of the building, subsidiary mains are connected to the ring mains, the subsidiary mains in turn supplying the loops that feed the radiators and hot-water supply in each block. Valves are arranged so that any subsidiary main or any loop can be shut off and emptied for cleaning or repairs—without interfering with the remainder of the system.

### Heating the Wards.

The wards are heated by radiators of the hospital type, with wide spacing between the loops to allow of easy access for cleaning.

Each radiator has its own valves, so that it may be cut out of the service at will and the rate of flow of water through it may be regulated. The radiators are enclosed in cases, especially designed by the architect to act as reservoirs. The cases consist of perforated cupboards. Provision has been made for cleaning the casings as well as the radiators. Fresh air is admitted from outside through louvred gratings placed behind the radiators. It is claimed that the casings act as reservoirs of warm air in cold weather. The ventilating gratings behind the radiators are 18 in. wide and 16 in. high; they are placed between the beds, and smaller gratings under each bed. There are two sets of hot-water pipes supplying the radiators on the two sides of each ward, one set of radiators being supplied from one set of pipes, and the other radiators supplied from the other set. The object is to prevent all of the radiators in any ward being off at the same time, the two sets of pipes being separately connected to the subsidiary mains or loops. It is claimed that the difference in temperature of the water leaving the heater and returning to it, after travelling through some miles of piping in the radiator system, is approximately 20° F. In the domestic hot-water supply system the difference of temperature necessarily varies with the quantity of water that is drawn off.

The temperature in the radiators is regulated at the heaters, at the different valves controlling the sub-mains and loops, and at the radiators themselves. It is necessarily higher in very cold weather than in moderate weather. In cold weather the temperature in the radiators may be as high as 200° F., while in very moderate weather it may be as low as 140° F.

Messrs. Dargue, Griffiths and Co., of Liverpool, fitted up the whole installation.

## Figures and Tables

**Figure f1:**